# Association of Interferon-gamma Induced Protein 10 Promoter Polymorphisms with the Disease Progression of Hepatitis B Virus Infection in Chinese Han Population

**DOI:** 10.1371/journal.pone.0072799

**Published:** 2013-09-04

**Authors:** Zhihui Xu, Yan Liu, Liming Liu, Xiaodong Li, Siyu Bai, Yihui Rong, Haibin Wang, Yuanli Mao, Shaojie Xin, Dongping Xu

**Affiliations:** 1 Viral Hepatitis Research Laboratory, Beijing 302 Hospital, Beijing, China; 2 Center for Clinical Laboratory, Beijing 302 Hospital, Beijing, China; 3 Medical Center for Liver Failure, Beijing 302 Hospital, Beijing, China; MOE Key Laboratory of Environment and Health, School of Public Health, Tongji Medical College, Huazhong University of Science and Technology, China

## Abstract

**Background and Aims:**

Interferon-gamma induced protein 10 (IP-10) was suggested to be involved in liver injury in viral hepatitis. This study aimed to investigate the impact of the single nucleotide polymorphisms (SNP) G-201A (rs1439490) in IP-10 gene on disease progression of hepatitis B virus (HBV) infection.

**Methods:**

The -201 SNP in IP-10 promoter was genotyped from 577 patients with different illness categories and 275 health controls; *In vitro* IP-10 promoter activity was compared between haplotype GG and AA homozygotes using luciferase reporter system in HepG2 cells. *In vivo* expression of IP-10 was compared between patients with -201 AA genotype and GG genotype.

**Results:**

The detected frequency of G-201A SNP was 17.8%, 25.3%, 26.6%, and 13.8% for patients with acute hepatitis B (AHB), patients with mild chronic hepatitis B (CHB-M), patients with severe chronic hepatitis B (CHB-S), and health controls, respectively. *In vitro* IP-10 promoter-driven luciferase activity in pGL3-Enhancer-201A transfected HepG2 cells was 1.43-fold higher than that in pGL3-Enhancer-201G transfected HepG2 cells (*P*<0.01). *In vivo* IP-10 transcriptional expression of peripheral blood mononuclear cells was 1.38-fold higher in patients with -201 AA genotype than in patients with -201 GG genotype (*P*<0.01).

**Conclusion:**

G-201A in promoter region of IP-10 gene was associated with liver disease progression in patients with HBV infection through up-regulating IP-10 expression.

## Introduction

Hepatitis B virus (HBV) is non-cytopathic itself and the liver damage caused by HBV infection is mainly immune-mediated [1]. The clinical resolution and disease progression of HBV infection are subsequent to the interaction between host immune responses and viral factors. Genetic susceptibilities such as some single nucleotide polymorphisms (SNPs) in human genes have been reported to be associated with viral clearance in HBV infection [2], [3], [4]. However, it is still not clear what human SNPs are involved and how they affect the advancement of HBV-related disease [5].

Interferon gamma (IFN-γ) was involved in both innate and adapted immune responses, and IFN-γ induces protein 10 (IP-10) is a cytokine that chemo-attracts T cells and NK cells that express CXCR3 ligands [6], [7]. Secreting by monocytes, endothelial cells, dendritic cells etc., IP-10 is widely involved in homing of immune cells, apoptosis, cells growth and angiostatic effects [8]. Particularly, serum IP-10 level was elevated in patients with viral hepatitis and elevated more in hepatitis C than in hepatitis B. IP-10 expression was correlated with histologic severity and lobular inflammation in patients with chronic hepatitis C virus infection [9]. Pre-treatment plasma level of IP-10 (cutoff: 600 pg/mL) could predict whether standard-of-care (interferon plus telaprevir) for hepatitis C would achieve a rapid viral response or sustained viral response [10]; the median serum IP-10 level in patients with spontaneous clearance of HCV was lower than that in patients without spontaneous clearance [11]. Expression of serum IP-10 and IL28B polymorphism was currently the best predictive marker combination for spontaneous clearance of the HCV [12]. For HBV infection, IP-10 expression was observed significantly lower than baseline in virological responders while was not observed in non-responders [13]. Serum IP-10 level was also significantly associated with the severity of hepatic inflammation, particularly in patients with only wild-type virus in another study. Limited studies were done with IP-10's impact on HBV infection in contrast to HCV infection.

The mechanism affecting IP-10's binding affinity to CXCR3 is not fully understood. In previous study, 21 SNPs was recognized by sequence analysis of the 4228 bp length IP-10 gene, and none of them was in the coding region [14]. Among the revealed SNPs, G-201A in the promoter region was located between the nuclear factor κβ1 and nuclear factor κβ2 binding sites and thus was picked out for investigation [14], [15]. The hypothesis of our study is that the variations in the promoter region may affect the immune response by altering the expression of IP-10 and further affect the disease progressions. Therefore, we investigated the allele frequencies of G-201A in group of acute hepatitis B (AHB), mild chronic hepatitis B (CHB-M) and severe chronic hepatitis B (CHB-S), and further conducted functional analysis of this SNP *in vitro* and *in vivo*.

## Materials and Methods

### Study subjects

The study population consisted of 577 unrelated Chinese HBV patients who visited Beijing 302 hospital and 275 healthy controls who donated at the blood donor center in Beijing 302 Hospital. The patients were categorized into AHB (196), CHB-M (193), and CHB-S (188) sub-groups with increasing disease severity. The categorization was based on the diagnostic criteria of 2000 Xi'an Viral Hepatitis Management Scheme issued by the Chinese Society of Infectious Diseases and Parasitology, and the Chinese Society of Hepatology of the Chinese Medical Association [16]. Briefly, the criteria for AHB were: no histories of HBV infection; acute onset of symptoms; and spontaneous HBsAg clearance within 6 months after the initial onset of illness. The criteria for CHB-M were: histories of HBV infection; persistence of HBsAg for at least 6 months; a histopathological diagnosis of mild liver injury with compatible laboratories or ultrasonographics. The criteria for CHB-S were: symptoms of severe liver injury; significant elevation of serum alanine aminotransferase (ALT). Confirmation of CHB-S diagnosis was made when one of the following was met: (1) serum albumin level >32 g/L; (2) serum total bilirubin (TBIL) >85.5 μmol/L; (3) plasma prothrombin activity (PTA) <60–40%; (4) serum cholinesterase <4500 IU/L. No evidence of cirrhosis, HCC, concomitant of HCV, HDV, or HIV infection, metastatic or autoimmune liver disease was found for any patients. The clinical backgrounds of patients and healthy controls were summarized (Table 1). This study was approved by the Ethics Committee of Beijing 302 Hospital. Written consents were obtained from all subjects.

**Table 1:** Clinical details of the studied population.

	HC (n = 275)	AHB (n = 196)	CHB-M (n = 193)	CHB-S (n = 188)	*P* value
Gender (M/F)	156/30	149/47	153/40	155/33	0.259
Age (years)	31.2±10.2	35.4±12.4	32.4±14.1	38.7±13.2	<0.001
Total bilirubin (µmol/L)	–	111.4±89.4	11.7±5.8	185.8±192.4	<0.001
Prothrombin activity (%)	–	95.1±24.9	93.5±19.7	81.6±20.0	<0.001
Albumin (g/L)	–	39.0±3.8	43.1±3.5	37.3±4.2	<0.001
Albumin/globulin	–	1.65±0.4	1.73±0.3	1.50±0.3	<0.001
Cholinesterase (IU/L)	–	5544.5±1746.6	7171.1±2324.0	4233.2±1548.8	<0.001
ALT (IU/L)	–	648.3±830.3	80.3±124.0	416.0±389.9	<0.001
HBV DNA (logcps/mL)	–	3.88±1.46	5.31±2.16	5.68±1.56	<0.001
HBeAg negative/positive	–	137/59	146/47	100/88	<0.001

HC, healthy controls; AHB, acute hepatitis B; CHB-M, mild chronic hepatitis B; CHB-S, severe chronic hepatitis B.

### Polymorphism genotyping

The SNPs G-201A polymorphisms in the 5′ flanking region of the IP-10 gene was the research interest. As the polymorphisms G-201A and C-1596T were in absolute LD (D′ = 1, r^2^ = 1, in a validation sample of 387) in previous study [14], G-201A genotypes were inferred from the C-1596T genotypes, whereas the determination for the latter is carried out by a cost-efficient method–the in-house nested polymerase chain reaction restriction fragment length polymorphism (PCR-RFLP) analysis. Namely, genomic DNA was extracted from 140 µL of serum using a DNAout kit (Tiandz Engineering, Beijing, China); the extracted DNA was dissolved in 80 µL of Tris-HCl buffer (0.1 mol/L, pH 8.0) and stored at −40°C before use. The primers for the first-round nested PCR were F (5′-CATCCTAGGATCAGGCTACTGT-3′) and R (5′- AGCTGACAACTTAGATACCAAC -3′); the primers for the second-round nested PCR were F (5′-GCAGATACTGTCTCAGAACCTGGTA-3′) and R (5′- TGTCACCATCTCTCATTTTGATTGT-3′). The first-round PCR consisted of a denaturing at 94°C for 3 min and 30 cycles at 94°C for 25 s, 52°C for 25 s, 72°C for 50 s. The second-round PCR consisted of a denaturing at 94°C for 3 min and 30 cycles at 94°C for 20 s, 54°C for 20 s, 72°C for 40 s. Amplicons of 499-base pair lengths were the PCR products, which were purified by QIAquick PCR purification kit (Qiagen, USA). After purification, 5 µL of the PCR products were digested within 15 U *Xba* I (Takara BioTech, Dalian, China) for the genotyping of C-1596T polymorphisms. Cleaved DNA fragments were identified by ultraviolet light after electrophoresis in 2% agarose gel stained by ethidium bromide. The homozygotes with C-1596T alleles yielded restriction fragments of 174/325 base pairs.

The accuracy of genotyping information for each -201A alleles that referred from PCR-RFLP analysis was validated by sequencing of 45% samples randomly from the studied population. G-201A polymorphisms were amplified by PCR method. The primers for the first-round PCR were F (5′-TCAAGGAGGACTGTCCAGGTAAATC-3′) and R (5′-TGGCAGTTTGATTCATGGTGC-3′), the second-round PCR were F (5′- GGAGGACTGTCCAGGTAAATCACTG-3′) and R (5′-TGAGGAATGTCTCAGAAAACGTGG-3′). The conditions for the first-round and second-round PCR amplifying G-201A were the same as that of amplifying C-1596T. Sequencing was performed using an ABI 3730xl DNA Analyzers (Applied Biosystems, Foster City, CA).

### Functional analyses *in vitro*

Genomic DNAs from haplotype GG and AA homozygotes of G-201A polymorphisms were chosen for reporter plasmid construction. DNA fragments corresponding to IP-10 promoter region from nucleotides −875 to +27 (relative to the first nucleotide of the open reading frame of IP-10) were amplified. The primers for the first-round PCR were F (5′-GAACCCCATCGTAAATCAACCTG-3′) and R (5′-CCTTGAATGCCACTTAGAGTCAG-3′); for the second-round PCR were F (5′- *CGGGGT ACC*CAAGGTCTGTCTCTATGCGTGC-3′) (introduced *Kpn* I as underlined) and R (5′-*CCCAAG CTT*GCAGCAAATCAGAATGGCAGTTTG-3′) (introduced *Hin*d III). The conditions for the first-round and second-round PCR were the same as described above. PCR products were purified by QIAquick PCR Purification Kit (Qiagen), after purification, the products were incubated with dATP and Taq DNA polymerase for adding A, and purified by a QIAquick Gel Extraction Kit. The ligation and transformation were performed according to the manufacturer's instructions of pGEM-T Vector System II (Promega Madison, WI, USA). The plasmids were extracted and digested with *Kpn* I and *Hin*d III, and the IP-10 promoter DNA fragments were purified by a QIAquick Gel Extraction Kit. The fragment was ligated to pGL3-Enhancer Vector and then transiently transfected HepG2 cells (Promega Madison, WI, USA). All reporter constructs and transformation was confirmed by direct sequencing after digestion with *Kpn* I and *Hin*d III.

HepG2 cells (3×10^5^) were divided into 6-well plates before transfection. Every well was transfected with 2 µg of one of the constructs together with 0.2 µg of pRL-TK vector (Promega), which served as the internal control for transfection efficiency, by FuGENE HD transfection reagent (Roche, USA). After 48 hours, cells were collected and the luciferase activities were measured using the Dual-Luciferase Reporter Assay system (Promega). The measure was performed in triplicate.

Whole bloods were obtained from 24 patients, including fifteen patients with AA genotype and nine patients with -201 GG genotype. Cell RNA was extracted from the peripheral blood mononuclear cells (PBMC) using the RNeasy Mini kit (Qiagen) according to the manufacturer's instructions. The IP-10 mRNA levels were determined by SYBR Green real-time PCR (Roche) with beta actin as the internal control. The primers for IP-10 were 5′- GCAGTTACACTAAAAGGTGACC -3′ and 5′-GGAAGCAGGGTCAGAACATC-3′, primers for beta actin were 5-GAGATGCGTTGTTACAGGAAG-3 and 5-CACGAAAGCAATGCTATCACC-3. The experiments were preformed in duplicate.

### Statistics

Statistical analysis was performed in SPSS (version 16.0; SPSS Inc, Chicago, IL). Chi-squared analysis was used to calculate the difference of G-201A genotype frequencies among groups. Cochran-Armitage test was used to examine the linear trend. Student *t* test was used to analyze the luciferase reporter assays and the transcriptional expression of IP-10 in G-201A and G-201G genotype *in vitro* and *in vivo*. *P* values less than 0.05 were considered as statistical significant.

## Results

### Frequency of G-201A among different groups

The percentages of -201A containing genotype at -201 were 17.8%, 25.3%, 26.6%, and 13.8% for patients with AHB, CHB-M, CHB-S, and healthy controls, respectively. The overall *P* value among four groups was 0.0037 by general *chi-* square test. Cochran-Armitage linear trend test (*P* = 0.0029, *Z* = −2.97) indicated an increasing trend of G-201A frequency among the groups (Table 2). Paired comparison showed significant differences in SNP frequency between HC *vs*. CHB-M, HC *vs*. CHB-S, AHB *vs*. CHB-S, and the odds ratio and *P* value were provided (Table 3). For the test consistence of SNP detection method, PCR-RFLP method showed a 98.7% identity with the results from direct PCR sequencing by Kappa test and indicated that the two methods were statistical consistent (*P*<0 .05).

**Table 2:** G-201A frequencies linearly increased among different HBV groups.

-201 Genotype	HC(n = 275) (%)	AHB (n = 196) (%)	CHB-M (n = 193) (%)	CHB-S (n = 188) (%)	*P* value
GG	237(86.2)	161(82.2)	144(74.7)	138(73.4)	<0.001
GA	35(12.7)	29(14.7)	46(23.7)	47(25.0)	<0.001
AA	3(1.1)	6(3.1)	3(1.6)	3(1.6)	
GA+AA	38(13.8)	35(17.8)	49(25.3).	50(26.6)	<0.001

HC, healthy controls; AHB, acute hepatitis B; CHB-M, mild chronic hepatitis B; CHB-S, severe chronic hepatitis B.

**Table 3:** G-201A frequencies and odds ratio for the association with disease progression.

Genotype	Control N (%)	Cases N (%)	OR (95% CI)	*P* value
**HC vs. AHB**				
**GG**	237 (86.2)	161(82.2)	1.0	
**GA**	35 (12.7)	29 (14.7)	1.22 (0.72–2.08)	0.463
**AA**	3 (1.1)	6 (3.1)	2.94 (0.73–11.94)	0.114
**GA+AA**	38 (13.8)	35(17.8)	1.36 (0.82–2.24)	0.233
**HC vs. CHB-M				
**GG**	237 (86.2)	144 (74.7)	1.0	
**GA**	35 (12.7)	46 (23.7)	2.16 (1.33–3.52)	0.002
**AA**	3 (1.1)	3 (1.6)	1.65 (0.33–8.26)	0.541
**GA+AA**	38 (13.8)	49 (25.3)	2.12 (1.32–3.40)	0.002
**HC vs. CHB-S				
**GG**	237 (86.2)	138 (73.4)	1.0	
**GA**	35 (12.7)	47 (25.0)	2.31 (1.42–3.75)	0.001
**AA**	3 (1.1)	3 (1.6)	1.72 (0.34–8.63)	0.342
**GA+AA**	38 (13.8)	50 (26.6)	2.26 (1.41–3.62)	0.001
**AHB vs. CHB-M**				
**GG**	161(82.2)	144(74.7)	1.0	
**GA**	29(14.7)	46(23.7)	1.77(1.06–2.97)	0.028
**AA**	6(3.1)	3(1.6)	0.56 (0.14–2.28)	0.41
**GA+AA**	35(17.8)	49(25.3)	1.57 (0.96–2.55)	0.071
* **AHB vs. CHB-S**				
**GG**	161(82.2)	138(73.4)	1.0	
**GA**	29(14.7)	47(25.0)	1.89 (1.13–3.17)	0.015
**AA**	6(3.1)	3(1.6)	0.58 (0.14–2.38)	0.48
**GA+AA**	35(17.8)	50(26.6)	1.67 (1.02–2.72)	0.039
**CHB-M vs CHB-S**				
**GG**	144(74.7)	138(73.4)	1	
**GA**	**46(23.7)**	**47(25.0)**	1.07 (0.67–1.70)	0.789
**AA**	3(1.6)	3(1.6)	1.04 (0.21–5.26)	0.959
**GA+AA**	**49(25.3)**	**50(26.6)**	1.07 (0.67–1.68)	0.798

* denotes *P*<0.05; **denotes *P*<0.01.

HC, healthy controls; AHB, acute hepatitis B; CHB-M, mild chronic hepatitis B; CHB-S, severe chronic hepatitis B.

### *In vitro* promoter-derived luciferase activity of pGL3-Enhancer-201A

**Figure 1:**
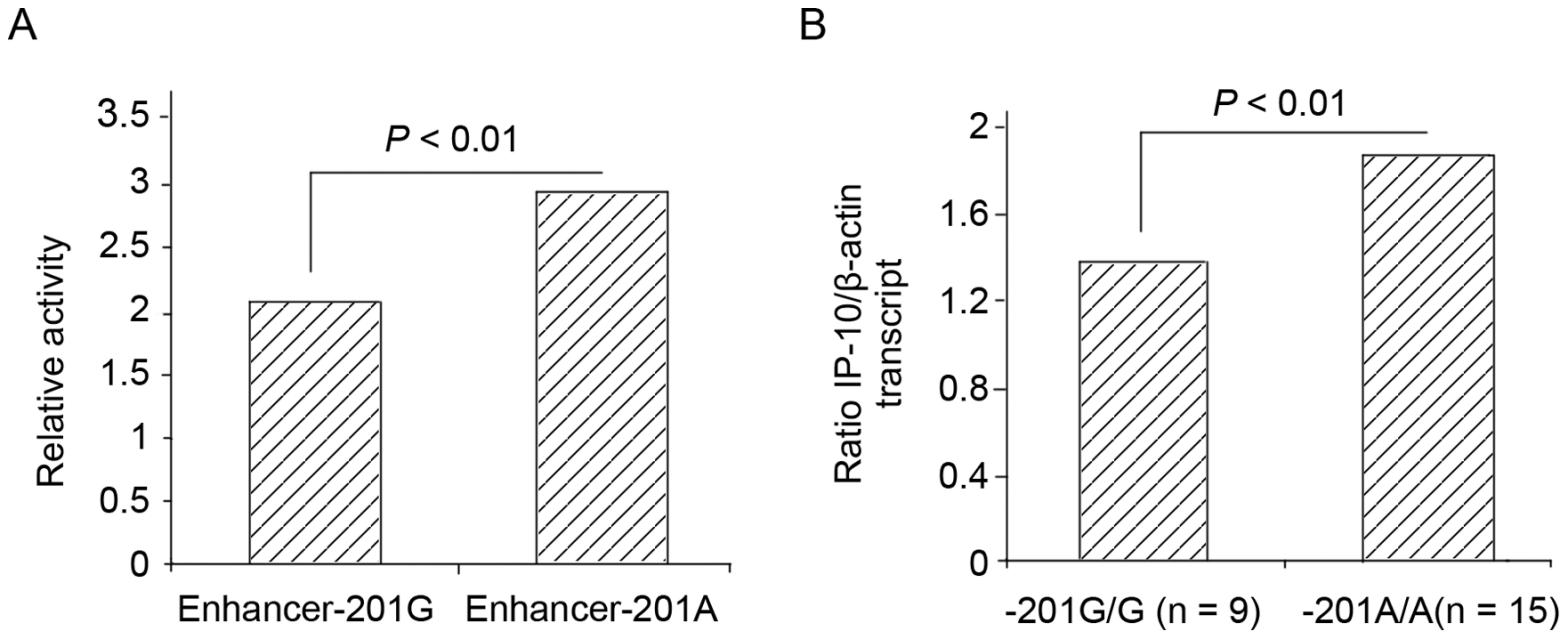
Correlation of IP-10 expression with the allele and genotype. (a) Luciferase activity in HepG2 cells transfected with promoter-reporter vectors corresponding to -201GG or AA haplotype homozygotes, respectively. The vertical axis represents the relative proportion of luciferase activity to corresponding pGL3-enhancer vectors. Data represent mean of representative data from three experiments performed in quadruplicate. *P* values were estimated by Student *t* test. (b) Expression of CXCL10 measured by quantitative SYBR PCR of RNAs purified from peripheral blood mononuclear cells of 24 patients. Columns, mean from triplicate measurements; bars, SD.

Genomic regions from nucleotides −875 to +27 of IP-10 promoter were cloned into pGL3-Enhancer vectors for luciferase activity measure. The inserted sequences of all reporter constructs were identical except nucleotides at site -201. The pGL3-Enhancer-201A constructs showed 1.43-fold higher relative luciferase activity than the pGL3-Enhancer-201G constructs (*P*<0.01) (Figure 1a), suggesting that the haplotype -201 AA was crucial for regulating the expression of IP-10. Data were obtained from three independent experiments, and each experiment was performed in quadruplicate.

### *In vivo* IP-10 level in PBMC from patients with -201 AA and GG genotype

The expression of IP-10 on transcriptional level in PBMC was measured by real-time PCR and compared between fifteen -201 AA patients and nine -201 GG patients at two independent sampling times. The ratio of IP-10 to β–actin transcript in PBMCs for -201 AA patients was 1.38 fold higher than that of -201 GG patients (*P*<0.01) (Figure 1b).

## Discussion

Many methods are practically available to detect SNPs, and the present study adapted a PCR-RFLP method for easy serum samples SNP detection. Comparing with the direct sequencing method, the PCR-RFLP method is fast, cost-effective, less laboratory demanding, thus favorable for study of large population. The assay was validated by direct sequencing for 45% samples and the two assays showed a 98.7% identity with statistical consistence.

A single SNP may cause a disease such as Mendelian disease, while a single SNP often function in coordination with other SNPs to manifest a disease condition as seen in osteoporosis and Hirschsprung's disease [17], [18]. The influence of genetic susceptibility in the development of CHB has been paid much attention. Previous studies verified SNPs at rs3077 and rs9277535 in HLA-DP region were chronic hepatitis B susceptible [19], while three HCC risk SNPs in KIF1B were not associated with progression to CHB [20]. In this study, we investigated the genetic susceptibility of G-201A in IP-10 for hepatitis B, and proposed a possible mechanism by conducting *in vitro* and *in vivo* functional comparison.

Elevation of IP-10 affects host immune responses to infection by altering activity of cells that expresses CXCR3 receptors [21], [22], [23]. For example, the crosstalk between T regulatory cells (Tregs) and natural killer T (NKT) cells was mediated by IP-10: when activated, the NKT cells secrete large amounts of IFN-γ, which induces the production of IP-10 and IP-10 chemoattract Tregs (which express CXCR3 receptors). In healthy organism, Tregs suppress the activation of effector T cell to maintain immune homostatus [24].However, in CHB, Tregs suppressed the immune response locally and contributed to the persistence of HBV infection in our previous observation [25]. Therefore, higher level of IP-10 means more recruited Tregs, especially within the liver, and suggests a stronger inhibition on immune responses. Particularly, the liver-derived Tregs express higher level of CXCR3 comparing with blood-derived Tregs [26], which make the inhibition even stronger.

Multiple factors may contribute to the elevation of IP-10. Decreased expression of CXCR3 receptor on plasmacytoid dendritic cells has been observed concomitant with an increased IP-10 level in plasma in patients with chronic HBV [27], suggesting that dysfunction or down-regulation of CXCR3 receptor may lead to the up-regulation of IP-10. In this study, we illustrated SNPs in IP-10 promoter could up-regulate the expression of IP-10 by altering its binding affinity and and therefore affect the disease progression. The A allele frequency was the lowest in healthy controls and the highest in patients with CHB-S. The linear increase trend of G-201A frequency from patients with AHB, to CHB-M and to CHB-S implied a predisposing role of this SNP. Functional analysis supported that allelic variation of G-201A enhanced IP-10 promoter activity *in vitro* and up-regulated the transcriptional expression of IP-10 *in vivo*. These results suggested that SNP at -201 played a role in how humans respond to the virus and progress the persistent infection.

However, we did not investigate potential SNPs outside IP-10 gene that co-work with G-201A, neither did we investigate other identified SNPs in IP-10 gene due to the consideration that the most frequent SNP C-1513T was equally presented between HBV non-progressed and progressed carriers in pervious study [14].

In summary, the current study explained the contribution of the G-201A in promoter region of IP-10 gene to the disease progression of CHB. Possible mechanisms of G-201A influencing the disease progression were altering immune responses through up-regulating IP-10 expression.

## References

[1] Dunn C, Brunetto M, Reynolds G, Christophides T, Lampertico P, et al (2007) HLA-DP and IL28B Polymorphisms: Influence of Host Genome on Hepatitis B Surface Antigen Seroclearance in Chronic Hepatitis B. J Exp Med. 204: 667–680.

[2] Liu Y, Zhang Y, Wen J, Liu L, Zhai X, et al (2012) A genetic variant in the promoter region of miR-106b-25 cluster and risk of HBV infection and hepatocellular carcinoma. PLoS One 7: e32230 doi: 10.1371/journal.pone.0032230 22393390 10.1371/journal.pone.0032230 PMC3290543

[3] Kimkong I, Tangkijvanich P, Hirankarn N (2013) Association of interferon-alpha gene polymorphisms with chronic hepatitis B virus infection. Int J Immunogenet in press. doi: 10.1111/iji.12055. 10.1111/iji.12055 23566196

[4] Guo X, Zhang Y, Li J, Ma J, Wei Z, et al (2011) Strong influence of human leukocyte antigen (HLA)-DP gene variants on development of persistent chronic hepatitis B virus carriers in the Han Chinese population. Hepatology 53: 422–428 doi: 10.1002/hep.24048 21274863 10.1002/hep.24048 PMC3056070

[5] Al-Qahtani A, Al-Anazi M, Viswan NA, Khalaf N, Abdo AA, et al (2012) Role of single nucleotide polymorphisms of KIF1B gene in HBV-associated viral hepatitis. PLoS One. 7: e45128 doi: 10.1371/journal.pone.0045128 10.1371/journal.pone.0045128 PMC3445584 23028799

[6] Dufour JH, Dziejman M, Liu MT, Leung JH, Lane TE, et al (2002) IFN-gamma-inducible protein 10 (IP-10; CXCL10)-deficient mice reveal a role for IP-10 in effector T cell generation and trafficking. J Immunol168: 3195–3204. 10.4049/jimmunol.168.7.3195 11907072

[7] Luster AD, Ravetch JV (1987) Biochemical characterization of a gamma interferon-inducible cytokine (IP-10). J Exp Med 166: 1084–1097. 2443596 10.1084/jem.166.4.1084 PMC2188708

[8] Liu M, Guo S, Stiles JK (2011) The emerging role of CXCL10 in cancer (Review). Oncol Lett 2: 583–589. 22848232 10.3892/ol.2011.300 PMC3406435

[9] Harvey CE, Post JJ, Palladinetti P, Freeman AJ, Ffrench RA, et al (2003) Expression of the chemokine IP-10 (CXCL10) by hepatocytes in chronic hepatitis C virus infection correlates with histological severity and lobular inflammation. J Leukoc Biol 74: 360–369. 12949239 10.1189/jlb.0303093

[10] Lagging M, Romero AI, Westin J, Norkrans G, Dhillon AP, et al (2006) IP-10 predicts viral response and therapeutic outcome in difficult-to-treat patients with HCV genotype 1 infection. Hepatology 44: 1617–1625. 17133471 10.1002/hep.21407

[11] Grebely J, Feld JJ, Applegate T, Matthews GV, Hellard M, et al.. (2013) Plasma interferon-gamma-inducible protein-10 (IP-10) levels during acute hepatitis C virus infection. Hepatology in press. doi: 10.1002/hep.26263. 10.1002/hep.26263 PMC3663887 23325615

[12] Beinhardt S, Aberle JH, Strasser M, Dulic-Lakovic E, Maieron A (2012) Serum level of IP-10 increases predictive value of IL28B polymorphisms for spontaneous clearance of acute HCV infection. Gastroenterology 142: 78–85 doi: 10.1053/j.gastro.2011.09.039 22192885 10.1053/j.gastro.2011.09.039

[13] Hou FQ, Wu XJ, Wang Y, Chen J, Liu YZ, et al.. (2013) Rapid downregulation of programmed death-1 and interferon-γ-inducible protein-10 expression is associated with favourable outcome during antiviral treatment of chronic hepatitis B. J Viral Hepat. Suppl 1: 18–26. doi: 10.1111/jvh.12060. 10.1111/jvh.12060 23458521

[14] Deng G, Zhou G, Zhang R, Zhai Y, Zhao W, et al (2008) Regulatory polymorphisms in the promoter of CXCL 10 gene and disease progression in male hepatitis B virus carriers. Gastroenterology 134: 716–726 doi: 10.1053/j.gastro.2007.12.044 18325387 10.1053/j.gastro.2007.12.044

[15] Majumder S, Zhou LZ, Chaturvedi P, Babcock G, Aras S, et al (1998) Regulation of human IP-10 gene expression in astrocytoma cells by inflammatory cytokines. J Neurosci Res 54: 169–180. 9788276 10.1002/(SICI)1097-4547(19981015)54:2<169::AID-JNR5>3.0.CO;2-C

[16] Chinese Society of Infectious Diseases and Parasitology, Chinese Society of Hepatology, of the Chinese Medical Association (2000) Management scheme of diagnostic and therapy criteria of viral hepatitis Zhonghua Gan Zang Bing Za Zhi Chin J Hepatol. 8: 324–329.

[17] Singh M, Singh P, Juneja PK, Singh S, Kaur T (2010) SNP–SNP interactions within APOE gene influence plasma lipids in postmenopausal osteoporosis. Rheumatolo Int 31: 421–423 doi:10.1007/s00296-010-1449-7 10.1007/s00296-010-1449-7 20340021

[18] Miao X, Leon T, Ngan E, So M, Yuan Z, et al (2010) Reduced RET expression in gut tissue of individuals carrying risk alleles of Hirschsprung's disease. Hum. Mol. Genet 19: 1461–7 doi:10.1093/hmg/ddq020 10.1093/hmg/ddq020 20089534

[19] Wang L, Wu X, Zhang W, Zhu D, Wang Y, et al (2011) Evaluation of genetic susceptibility loci for chronic hepatitis B in Chinese: two independent case-control studies PLoS One. 6(3): e17608 doi:10.1371/journal.pone.0017608 10.1371/journal.pone.0017608 PMC3050917 21408128

[20] Zhong R, Tian Y, Liu L, Qiu Q, Wang Y, et al (2012) HBV-related hepatocellular carcinoma susceptibility gene KIF1B is not associated with development of chronic hepatitis B. PLoS ONE. 7(2): e28839 doi:10.1371/journal.pone.0028839 10.1371/journal.pone.0028839 PMC3283615 22363396

[21] Berenguer J, Fernandez-Rodríguez A, Jimenez-Sousa MA, Cosín J, Zarate P, et al (2012) High plasma CXCL10 levels are associated with HCV-genotype 1, and higher insulin resistance, fibrosis, and HIV viral load in HIV/HCV coinfected patients. Cytokine 57: 25–29 doi: 10.1016/j.cyto.2011.10.020 22136974 10.1016/j.cyto.2011.10.020

[22] Bayard F, Godon O, Nalpas B, Costentin C, Zhu R, et al (2012) T-cell responses to hepatitis B splice-generated protein of hepatitis B virus and inflammatory cytokines/chemokines in chronic hepatitis B patients. ANRS study: HB EP 02 HBSP-FIBRO. J Viral Hepat 19: 872–880 doi: 10.1111/j.1365-2893.2012.01611.x 23121366 10.1111/j.1365-2893.2012.01611.x

[23] Ismail N, Walker DH, Ghose P, Tang YW (2012) Immune mediators of protective and pathogenic immune responses in patients with mild and fatal human monocytotropic ehrlichiosis. BMC Immunol 13: 26 doi: 10.1186/1471-2172-13-26 22607204 10.1186/1471-2172-13-26 PMC3517396

[24] Oo YH, Weston CJ, Lalor PF, Curbishley SM, Withers DR, et al (2010) Distinct roles for CCR4 and CXCR3 in the recruitment and positioning of regulatory T cells in the inflamed human liver. J Immunol 184: 2886–2898 doi: 10.4049/jimmunol.0901216 20164417 10.4049/jimmunol.0901216

[25] Xu D, Fu J, Jin L, Zhang H, Zhou C (2006) Circulating and Liver Resident CD4+CD25+ Regulatory T Cells Actively Influence the Antiviral Immune Response and Disease Progression in Patients with Hepatitis B. J Immunol. 177: 739–947. 10.4049/jimmunol.177.1.739 16785573

[26] Santodomingo-Garzon T, Han J, Le T, Yang Y, Swain MG (2009) Natural killer T cells regulate the homing of chemokine CXC receptor 3-positive regulatory T cells to the liver in mice Hepatology. 49: 1267–1276. 10.1002/hep.22761 19140218

[27] Martinet J, Dufeu-Duchesne T, Bruder Costa J, Larrat S, Marlu A, et al (2012) Altered functions of plasmacytoid dendritic cells and reduced cytolytic activity of natural killer cells in patients with chronic HBV infection. Gastroenterology 143: 1586–1596 doi: 10.1053/j.gastro.2012.08.046 22960656 10.1053/j.gastro.2012.08.046

